# Job demands and resources in relation to burnout and educational coexistence among Chilean education workers

**DOI:** 10.3389/fpsyt.2026.1844349

**Published:** 2026-06-18

**Authors:** Ricardo Jorquera-Gutierrez, Pedro R. Gil-Monte, Pedro Gil-LaOrden

**Affiliations:** 1 Department of Psychology, Universidad de Atacama, Copiapó, Chile; 2 Unidad de Investigación Psicosocial de la Conducta Organizacional (UNIPSICO), Department of Social Psychology, Universitat de València, Valencia, Spain

**Keywords:** burnout, educational coexistence, psychosocial risks, quality of work life, teachers

## Abstract

This study aimed to examine the fit of a structural equation model relating selected job demands and job resources to burnout and perceptions of educational coexistence among Chilean education personnel. A total of 710 workers from this sector (administrators, teachers, and teaching assistants) participated. Structural equation modeling (SEM) was conducted using robust maximum likelihood estimation and absolute and incremental fit indices. The proposed model showed acceptable fit (χ^²^ = 3181, df = 1307, p <.001; CFI _Robust_ = 0.911; TLI _Robust_ = 0.907; GFI = 0.956; RMSEA _Robust_ = 0.049). Workload and work-family conflict were positively associated with burnout (R^²^ = 0.612; p <.001), whereas role clarity and social support were positively associated with perceptions of educational coexistence (R^²^ = 0.396; p <.001). Overall, the findings are consistent with the Job Demands-Resources framework and suggest that, in this sample, job demands are more strongly linked to burnout, whereas job resources are more strongly linked to perceptions of educational coexistence. The study is discussed in relation to the Chilean educational context and the implementation of the National Policy on Educational Coexistence.

## 1 Introduction

The issue of mental health in Chile has gained significant prominence in recent years. Statistics from 2024 show that sick leave due to mental health issues accounts for 33.1% of all sick leave (1). By gender, women are the most affected (65% of sick leave due to mental health issues), and by age, the population between 25 and 44 years old accounts for 61% of these cases.

In the education sector, teachers’ mental health has garnered increased attention due to the challenges posed by the COVID-19 pandemic and the demands of the education system. Some studies (2, 3) reported that 58% of teachers had mental health problems, highlighting associations with working in subsidized private schools, unpaid overtime, and absenteeism due to illness. A relationship was also found between mental health and life satisfaction, identifying protective factors such as the use of socio-emotional strategies and levels of spirituality (4).

One of the mental health issues that has garnered growing interest is burnout. Burnout is a psychological syndrome that arises as a prolonged response to chronic work-related stress, particularly in service professions, and is characterized by a progressive deterioration in the worker’s emotional, cognitive, and attitudinal well-being (5, 6). It has been defined by three main dimensions: emotional exhaustion, understood as a feeling of being physically and emotionally drained; cynicism or depersonalization, referring to negative attitudes and detachment toward work or the people being served; and professional ineffectiveness or a perception of lack of achievement and competence at work (7–9). This model by Maslach and Jackson has been the most influential and widely used in international research.

Gil-Monte (6, 10 proposed an alternative model, particularly relevant in the Spanish-speaking context, which expands the traditional conceptualization by including a fourth dimension and nuances in the burnout development process (11). This model includes enthusiasm toward the job, understood as the desire to achieve work goals as a source of personal satisfaction; psychological exhaustion or emotional and physical exhaustion from daily interaction with people who have problems; indolence, referring to negative attitudes, indifference, and cynicism toward users or clients; and guilt, which are feelings of guilt arising from negative attitudes developed at work (6, 11).

Gil-Monte’s model, operationalized through the Spanish Burnout Inventory (SBI) (12, 13), emphasizes the role of guilt as a distinguishing symptom and mediator between burnout and its health consequences, allowing for the identification of profiles and developmental trajectories of the syndrome (5, 11, 14).

Although burnout is a significant and well-documented problem among teachers and other education workers in Chile, the literature on the subject remains limited at the national level.

The literature highlights that public and municipal schoolteachers have reported moderate to high levels of burnout, particularly in terms of emotional exhaustion (up to 34% in some studies) (15–17). Meanwhile, factors such as work overload, precarious conditions, lack of resources and institutional support, and job insecurity are among those observed to contribute significantly to burnout (15, 18, 19). At the same time, protective factors such as social support and job satisfaction (16), teacher resilience, self-care and institutional support, intrinsic motivation, and experience (20, 21) have also been identified.

In addition, from a role perspective, burnout affects both classroom teachers and administrators and support staff, although most studies focus on teachers (17, 18, 22).

One of the most widely used frameworks for understanding occupational well-being is the Job Demands-Resources (JD-R) theory. This theory proposes that every occupation involves a configuration of demands and resources that can be organized into two broad processes. Job demands are those physical, psychological, social, or organizational aspects of work that require sustained effort and are therefore associated with physiological and psychological costs. Job resources are those aspects of work that facilitate goal attainment, reduce the impact of demands, and support learning, development, and motivation (23–26). In this sense, JD-R offers a general framework for examining how working conditions are related to worker well-being and organizationally relevant outcomes.

Within this framework, a health-impairment process is expected when high job demands consume workers’ energy and are associated with strain outcomes such as burnout. A motivational process is expected when job resources foster more adaptive functioning and are associated with more positive evaluations of work and organization. Importantly, JD-R theory does not require that all resources relate only to motivational states or that all demands relate only to health impairment; rather, it provides a flexible theoretical architecture for testing whether specific demands and resources are more closely linked to particular outcomes in a given occupational context (23–25).

In the present study, the JD-R theory is used as the overarching theoretical framework, whereas the UNIPSICO battery is used as the operational system for selecting and measuring specific psychosocial demands and resources. The UNIPSICO model developed by Gil-Monte (27, 28 is conceptually consistent with JD-R and organizes psychosocial working conditions into demand factors and resource factors. Among demands, it includes workload, interpersonal conflict, inequity in social exchange, role conflict, and role ambiguity. Among resources, it includes social support at work, availability of resources, feedback, and autonomy. Thus, in this manuscript, JD-R provides theoretical logic and UNIPSICO provides the measurement strategy for selected psychosocial conditions.

Burnout, in turn, was examined through Gil-Monte’s framework rather than through the Maslach model. This decision was based on both conceptual and contextual considerations. Conceptually, Gil-Monte’s model preserves the central idea of burnout as a response to chronic occupational stress but distinguishes enthusiasm toward the job, psychological exhaustion, indolence, and guilt, thereby incorporating the moral and interpersonal consequences of work in human-service occupations. Contextually, this framework has been extensively developed and applied in Spanish-speaking settings, and the Spanish Burnout Inventory has shown adequate psychometric performance in Chilean samples (6, 10, 12, 13, 29). Therefore, the present study does not treat JD-R, UNIPSICO, and Gil-Monte’s model as competing explanations; instead, JD-R is used to explain expected patterns of association, UNIPSICO to assess selected demands and resources, and Gil-Monte’s framework to represent burnout as the strain-related outcome.

From this perspective, the study examines whether selected job demands are more strongly related to burnout and whether selected job resources are more strongly related to perceived educational coexistence among Chilean education workers.

A second key construct in this study is educational coexistence. This concept refers broadly to the quality of everyday relationships, participation, care, inclusion, conflict management, and democratic life within educational communities. In Chile, educational coexistence is not limited to school discipline or the absence of violence; rather, it is treated as a formative and organizational process that shapes how members of the school community interact and how institutions create conditions for respectful participation, learning, and collective well-being. This perspective has been reinforced in the updated National Policy on Educational Coexistence (PNCE) 2024–2030 (30), which includes a national action plan and guidance materials for schools and explicitly links coexistence with socioemotional development, mental health, inclusion, and school management.

In the current Chilean policy framework, educational coexistence is organized around five dimensions (30): an ethical dimension centered on collective care and inclusion; an educational or formative dimension that treats coexistence as something that is taught and learned; dimensions referring to ways of coexisting in everyday interactions; learning contexts that include institutional, social, and territorial conditions; and a management dimension focused on strategies, coordination, and prevention. These dimensions support the view that workers’ perceptions of coexistence are likely to depend not only on student behavior or disciplinary events, but also on organizational resources such as clear roles, coordination, and supportive professional relationships. This theoretical link is especially relevant in schools, where the implementation of coexistence policies depends on coordinated staff action, shared expectations, and support across occupational roles.

Given these theoretical frameworks, the hypotheses of this study are as follows:

H1: Workload will be positively and significantly related to burnout.
H2: Work-Family Conflict will be positively and significantly related to burnout.
H3: Social Support will be positively and significantly related to Perceived Educational Coexistence.
H4: Role Clarity will be positively and significantly related to Perceived Educational Coexistence.

**Figure 1:**
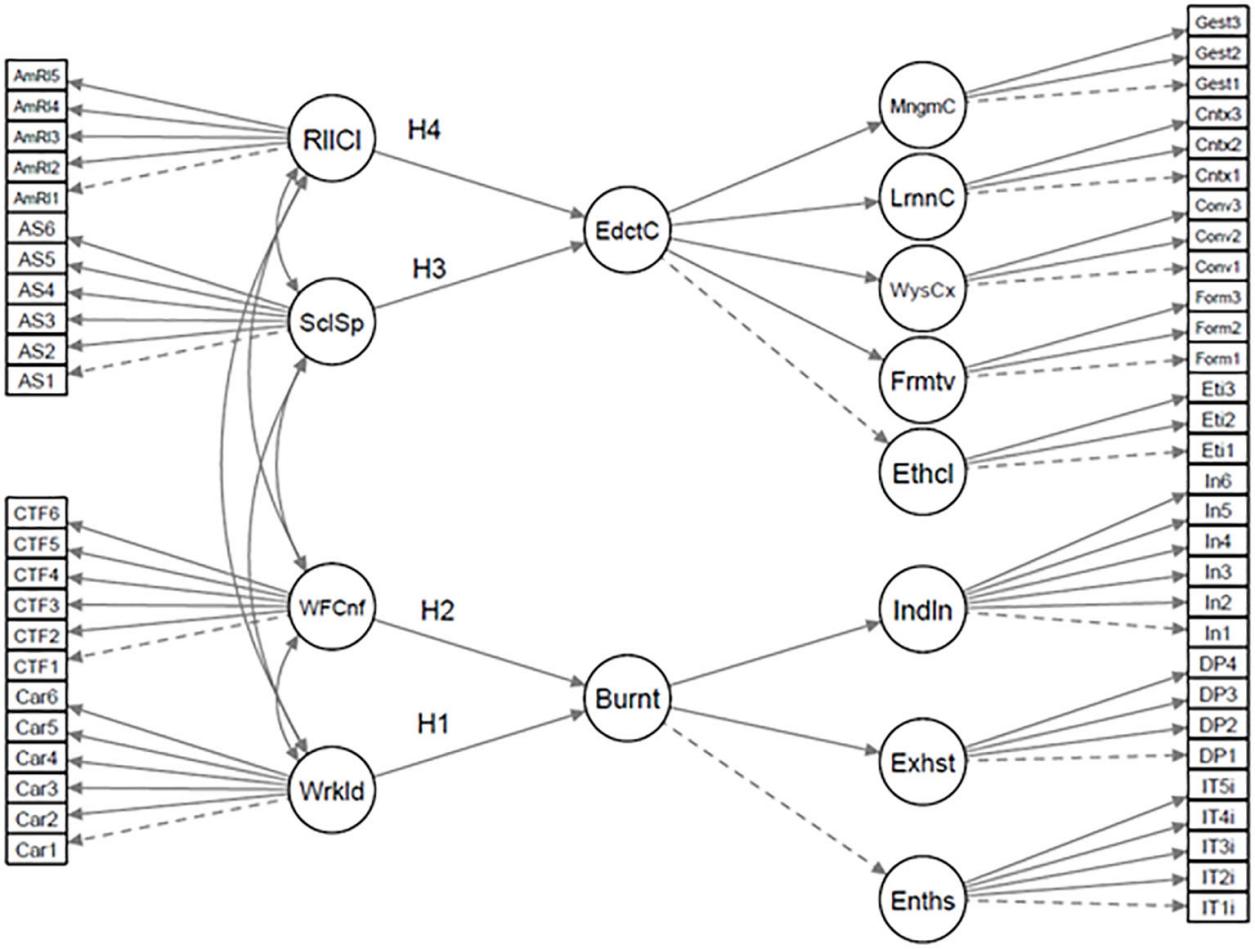
Hypothetical structural model of the relationship between psychosocial factors, burnout, and educational coexistence among education sector workers.

These hypotheses are based on previous research indicating that certain job demands are consistently associated with burnout, while specific job resources are associated with more positive perceptions of the school environment and social functioning. The present study evaluates a structural equation model in which workload and work-family conflict are positively associated with burnout, and social support and role clarity are positively associated with perceived educational coexistence (see **Figure 1**).

Studies consistently show that higher workload (administrative tasks, time pressure, and emotional demands) is associated with burnout, particularly in the dimensions of emotional exhaustion and depersonalization (31–36).

Work-family conflict is also one of the most consistent correlates of burnout among teachers. Two directions are usually distinguished: work-to-family and family-to-work, with the former tending to show a stronger association (37, 38), suggesting that work demands may be more closely related to interference with family life and to higher levels of exhaustion in this occupational group.

When roles within the school are clearly defined and consistent, the school climate and relationships may be perceived more favorably. In this regard, a teacher’s conciliatory and proactive stance, together with clear authority, has been associated with greater acceptance and respect among students and with more positive classroom and school climate indicators (39–42).

Similarly, there is evidence that social support among staff members and from the institution is an important resource for well-being. Supportive relationships with colleagues have been associated with greater well-being, stronger professional ties, and more favorable indicators of teaching practice and organizational functioning (43). Furthermore, high levels of teacher support are associated with more prosocial behaviors among students, whereas teacher conflict is associated with higher levels of student aggression (44).

## 2 Materials and methods

### 2.1 Research design

A non-experimental, cross-sectional design with a correlational scope was employed. Data were collected at one point in time to examine the statistical associations between psychosocial working conditions, burnout, and perceptions of educational coexistence.

### 2.2 Participants

The sample consisted of 710 education sector workers from 35 educational institutions in the Atacama Region, Chile. By role, 331 (46.6%) were teachers, 322 (45.4%) were teaching assistants, and 57 (8%) were administrators. By gender, 559 (78.7%) were women, 148 (20.8%) were men, and 3 (0.4%) identified as other. Their average age was 42.9 years, with a range extending from 20 to 76 years.

Non-probability convenience sampling was used due to the accessibility of educational institutions. The inclusion criteria were: (1) working in an educational institution in the Atacama Region at the time of the study and (2) serving in a role as a teacher, teaching assistant, or administrator.

### 2.3 Instruments

Three instruments were used for data collection:

1. Spanish Burnout Inventory (SBI) (12). This instrument consists of 20 items rated on a 5-point frequency scale (0 = “Never” to 4 = “Very frequently: every day”). It assesses four dimensions: Enthusiasm towards the job: The individual’s desire to achieve work goals (5 items); Psychological exhaustion: Emotional and physical exhaustion (4 items); Indolence: Negative attitudes toward students (6 items); Guilt: Feelings of remorse regarding work behavior (5 items). The overall burnout score is obtained by averaging the Exhaustion, Indolence, and Guilt scales (6). Its psychometric properties have been validated among Chilean education professionals (13, 29).
2. UNIPSICO Battery (Psychosocial Factors): Specific subscales from the UNIPSICO Battery (27, 28) were selected to measure Demands and Resources. Regarding work demands, the Work-Family Conflict and Workload scales were used. Regarding resources, the Social Support and Role Clarity scales were used. These scales employ a 5-point Likert-type response system. Previous studies in Spanish-speaking populations report internal consistency indices (Cronbach’s alpha) above.70 for these subscales.
3. Questionnaire on Perceptions of Educational Coexistence: For this study, an *ad hoc* scale was developed based on the dimensions proposed in Chile’s National Policy on Educational Coexistence (30). The instrument was designed to capture workers’ perceptions of how the principles of this policy are expressed in the operation of their educational institutions. The items were developed through a theory-based, top-down procedure using the policy framework as the main reference. The instrument comprises 15 items distributed across five dimensions (three items per dimension): (a) ethical dimension (e.g., “In my educational community, reflection on the values and ethical principles that guide school coexistence is encouraged”), (b) formative dimension (e.g., “In my educational community, one learns to live together through daily interaction in all educational spaces”), (c) ways of coexistence (e.g., “In my educational community, relationships are based on collective care for all”), (d) learning contexts (e.g., “In my educational community, care is taken to maintain an adequate classroom climate based on good treatment and trust”), and (e) management of coexistence (e.g., “In my educational community, educational coexistence is approached from a preventive and formative perspective”). Items were rated on a 7-point Likert scale, with higher scores indicating more favorable perceptions of educational coexistence. A preliminary analysis of a subsample of 376 participants showed that the measure fit a second-order model acceptably (χ^2^ = 121, df = 85, p = 0.007; CFI = 0.989, TLI = 0.986, RMSEA = 0.033) and had high internal consistency (α ≥ 0.896 across factors). In the full sample, the first-order dimensions demonstrated evidence of reliability and convergent validity, with omega coefficients ranging from 0.890 to 0.943 and average variance extracted (AVE) values ranging from 0.730 to 0.848.

### 2.4 Procedures

The research was conducted within the context of a specialized diploma program on educational coexistence. First, institutional authorization was obtained from the participating schools through formal letters addressed to school administrators. Then, a designated representative from each institution informed potential participants about the study’s objectives, academic purpose, voluntary participation, and exclusive scientific use of the collected data.

Data was collected through an online, self-administered survey hosted on Google Forms. The landing page of the questionnaire presented a digital informed consent form that described the study purpose, the voluntary nature of participation, the right to withdraw at any time before submission, and the procedures used to protect confidentiality.

The study was observational, noninvasive, and anonymous. No direct personally identifiable information was requested or recorded, and responses were analyzed only in aggregate form. Access to the dataset was restricted to the research team, and data management procedures followed Chilean Law No. 19,628 on the protection of personal data.

Under the applicable local and institutional framework, this non-interventional academic study did not require formal review by an institutional ethics committee. This exemption from formal review did not alter the ethical safeguards applied in the study, which included informed consent, voluntary participation, anonymity, confidentiality, restricted data access, and data protection procedures.

### 2.5 Data analysis

Data analysis was performed using structural equation modeling (SEM). The analyses were conducted in Jamovi 2.6.44 using robust maximum likelihood estimation (MLR). MLR was selected because the descriptive analyses suggested non-normality in several observed variables. Given these distributional characteristics, robust estimation was considered appropriate because it provides standard errors and fit statistics adjusted for non-normality while retaining the advantages of maximum likelihood estimation. Model evaluation considered both measurement and structural components. The reported fit indices were chi-square (χ^2^), Tucker-Lewis Index (TLI), Comparative Fit Index (CFI), Goodness-of-Fit Index (GFI), and Root Mean Square Error of Approximation (RMSEA). Acceptable fit was indicated by values above 0.90 for CFI, TLI, and GFI, as well as values below 0.06 for RMSEA (45, 46). Descriptive statistics, intercorrelations, and internal consistency/convergent validity indicators were also examined. In addition to Cronbach’s alpha, McDonald’s omega coefficients (ω) and average variance extracted (AVE) were calculated for the first-order constructs. Finally, multigroup invariance of the model by role (administrator, teaching assistant, or teacher) was explored using robust χ^2^, robust CFI, and robust RMSEA across configural, metric, and scalar models. Changes smaller than 0.010 in CFI and smaller than 0.015 in RMSEA were used as descriptive reference criteria (47).

## 3 Results

The reliability results indicate acceptable to high score consistency across the first-order scales used in the study. Cronbach’s alpha ranged from 0.73 (Role Clarity) to 0.95 (Ways of Coexistence), and the omega coefficients showed a very similar pattern (ω = 0.75–0.94). Average variance extracted (AVE) ranged from 0.40 (Role Clarity) to 0.85 (Ways of Coexistence). In this context, the AVE values below the conventional 0.50 threshold for Role Clarity and Social Support suggest that convergent validity for these constructs should be interpreted with caution, even though their internal consistency indices were acceptable.

The descriptive results show a mean of 5.39 (SD = 1.37) for educational coexistence on a scale of 1 to 7. The resource variables show a mean of 3.31 (SD = 0.73) for role clarity and 2.60 (SD = 0.89) for social support. Meanwhile, the demand variables show a mean of 1.72 (SD = 0.84) for workload and 0.77 (SD = 0.73) for work-family conflict. Regarding the burnout dimensions, the highest means are found in enthusiasm towards the job (M = 3.04; SD = 0.85) and the lowest in indolence (M = 0.57; SD = 0.63). The overall burnout score shows a mean of 0.97 (SD = 0.62).

Regarding skewness, values were negative for positive value and/or resource variables (educational coexistence, role clarity, social support, and enthusiasm toward the job) and positive for demand variables, burnout, and the dimensions of psychological exhaustion and indolence. Several skewness coefficients were close to or above |1|, particularly for role clarity, work-family conflict, and indolence. Kurtosis showed more pronounced departures from normality: role clarity (Ku = 12.83), work-family conflict (Ku = 21.23), and especially indolence (Ku = 41.10) exhibited substantial leptokurtosis rather than only slight deviations. These distributional features were considered in the analyses using robust maximum likelihood estimation (see **Table 1**).

**Table 1:** Descriptive statistics, Cronbach’s alpha, and correlations among the study variables.

	Mean	SD	Sk	Ku	α	ω	AVE	1	2	3	4	5	6	7	8	9	10	11	12	13
1. Educational Coexistence	5.39	1.37	-0.77	-0,06	0.98	0.98		—												
2. Ethical	5.25	1.62	-0.71	-0.52	0.89	0.89	0.73	0.92***	—											
3. Formative	5.48	1.42	-0.85	0.05	0.92	0.92	0.79	0.95***	0.87***	—										
4. Ways Coexistence	5.09	1.57	-0.64	-0.36	0.94	0.94	0.85	0.94***	0.86***	0.89***	—									
5. Learning Context	5.54	1.34	-0.92	0.38	0.91	0.91	0.77	0.94***	0.81***	0.89***	0.87***	—								
6. Management Coexistence	5.48	1.42	-0.92	0.32	0.93	0.93	0.81	0.93***	0.81***	0.85***	0.84***	0.88***	—							
7.Role Clarity	3.31	0.73	-1.24	12.83	0.73	0.75	0.40	0.49***	0.48***	0.45***	0.46***	0.43***	0.45***	—						
8. Social Support	2.60	0.89	-0.33	-0.51	0.84	0.84	0.48	0.48***	0.49***	0.45***	0.47***	0.43***	0.41***	0.40***	—					
9. Workload	1.72	0.84	0.18	-0.31	0.84	0.84	0.47	-0.31***	-0.26***	-0.26***	-0.32***	-0.28***	-0.28***	-0.22***	-0.19***	—				
10. Work-Family Conflict	0.77	0.73	1.29	21.23	0.86	0.87	0.54	-0.23***	-0.19***	-0.19***	-0.23***	-0.20***	-0.25***	-0.15***	-0.19***	0.60***	—			
11. Enthusiasm towards the job	3.04	0.85	-0.90	0.50	0.81	0.81	0.46	0.46***	0.43***	0.43***	0.43***	0.43***	0.41***	0.33***	0.42***	-0.12***	-0.12**	—		
12. Psychological exhaustion	1.58	1.11	0.43	-0.66	0.91	0.91	0.71	-0.31***	-0.26***	-0.25***	-0.34***	-0.29***	-0.30***	-0.23***	-0.29***	0.61***	0.63***	-0.25***	—	
13. Indolence	0.57	0.63	1.76	41.10	0.78	0.79	0.41	-0.29***	-0.26***	-0.27***	-0.27***	-0.28***	-0.27***	-0.15***	-0.22***	0.29***	0.30***	-0.32***	0.44***	—
14. Burnout	0.97	0.62	0.81	0.66	0.85	0.86		-0.47***	-0.43***	-0.43***	-0.46***	-0.44***	-0.44***	-0.32***	-0.42***	0.47***	0.47***	-0.71***	0.77***	0.76***

*p <.05, **p <.01, ***p <.001.

At the bivariate level, educational coexistence was positively correlated with role clarity (r = 0.49), social support (r = 0.48), and job enthusiasm (r = 0.46). It was also negatively correlated with workload (r = -0.31), work-family conflict (r = -0.23), psychological exhaustion (r = -0.31), indolence (r = -0.29), and the overall burnout score (r = -0.47). These patterns are consistent with those observed at the level of specific components of educational coexistence (see **Table 1**).

The overall model fit was evaluated. The chi-square statistic (χ² = 3181, df = 1307, p <.001) was significant. The incremental fit indices (CFI, TLI, and GFI) were within acceptable and adequate ranges (CFI Robust = 0.911; TLI Robust = 0.907; GFI = 0.956). The Root Mean Square Error of Approximation (RMSEA Robust = 0.049) was optimal.

**Figure 2:**
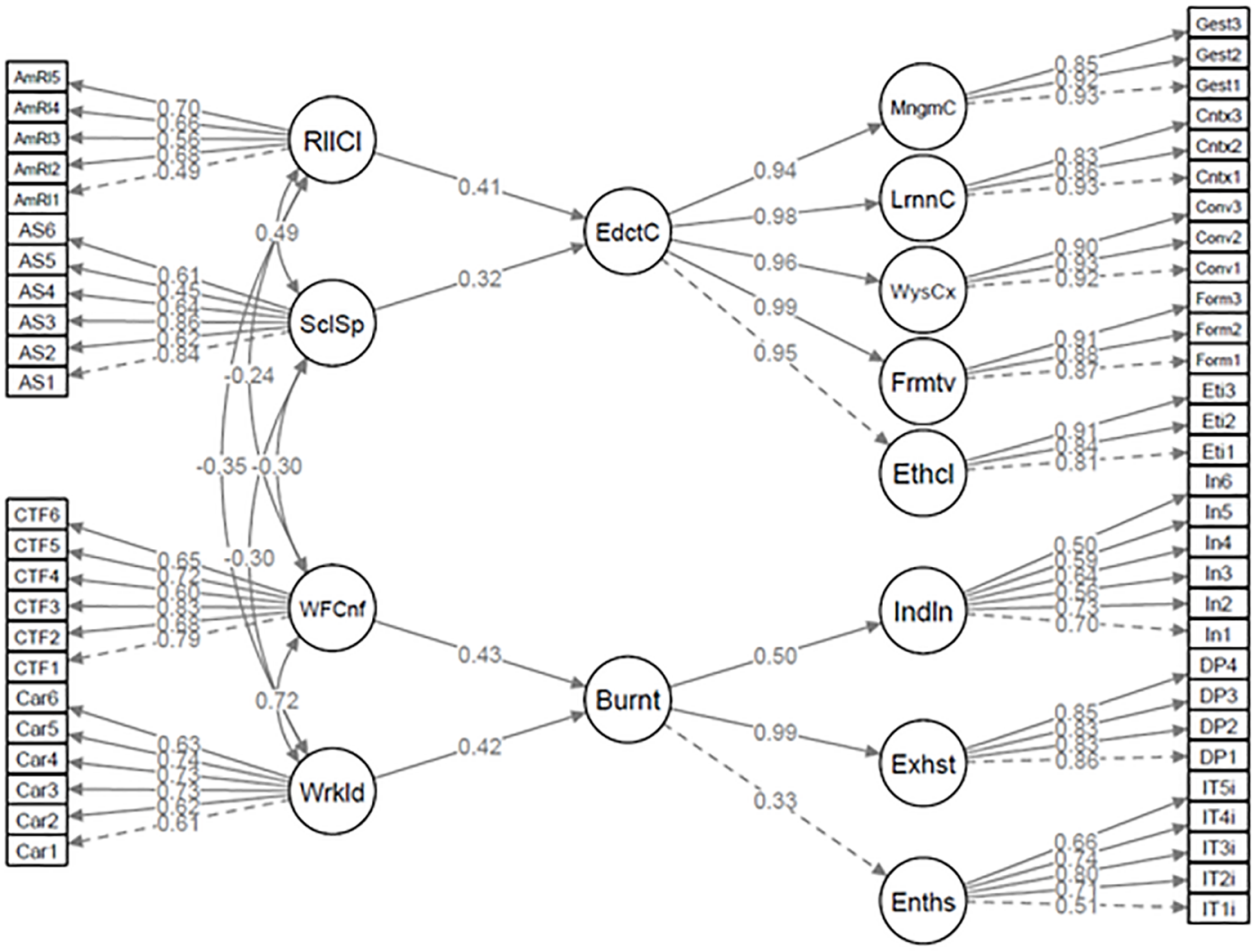
Structural model of the relationship between psychosocial factors, burnout, and educational coexistence among education sector workers.

Upon examining the correlations between the exogenous variables (workload, work-family conflict, social support, and role clarity), it was found that all were significant (p <.001). The correlation between the two demand variables—workload and work-family conflict—was strong and positive (β = 0.72). The same holds for the resource variables. The correlation between social support and role clarity was positive (β = 0.49) (**Figure 2**).

It is important to note that there are significant negative correlations between the demand variables and the resource variables. Workload correlates negatively with both social support (β = -0.30) and role clarity (β = -0.35). Work-family conflict, meanwhile, exhibits the same pattern with social support (β = -0.30) and role clarity (β = -0.24).

The structural model examined the extent to which the selected demands were associated with burnout and the selected resources were associated with educational coexistence. The model accounted for 61.2% of the variance in burnout (R² = 0.61) and 39.6% of the variance in educational coexistence (R² = 0.40).

Work-family conflict (β = 0.43, p <.001) and workload (β = 0.42, p <.001) were positively associated with burnout. Similarly, role clarity (β = 0.41, p <.001) and social support (β = 0.32, p <.001) were positively associated with educational coexistence.

A multigroup invariance analysis was conducted to examine whether the model showed a similar pattern across participant roles (administrators, teaching assistants, and teachers). The configural model showed limited fit (CFI_Robust_ = 0.836; RMSEA_Robust_ = 0.069), which suggests caution when interpreting the subsequent invariance steps. Within that context, the metric model did not show meaningful deterioration relative to the configural model (ΔCFI = -0.001; ΔRMSEA = 0.000), and the scalar model also remained within conventional change thresholds (ΔCFI = -0.010; ΔRMSEA = 0.001). Taken together, these results may be viewed as exploratory evidence of broadly comparable patterns across roles, rather than as strong confirmation of scalar invariance (see **Table 2**).

**Table 2:** Factor invariance by role.

	X2	DF	X2/DF	CFI Robust	RMSEARobust	ΔCFI	ΔRMSEA
Configural Invariance	8559	3921	2.183	0.836	0.069		
Metric Invariance	8734	4015	2.175	0.835	0.069	-0.001	0.000
Scalar Invariance	9080	4093	2.218	0.825	0.070	-0.010	0.001

## 4 Discussion

The findings are consistent with the Job Demands-Resources (JD-R) framework (23, 26) as an organizing perspective for understanding the relationship between selected psychosocial working conditions and burnout and educational coexistence in this sample of Chilean educators. The results indicate that the observed pattern of associations follows the general expectation of the JD-R theory: job demands are more strongly related to burnout, while job resources are more strongly related to perceptions of educational coexistence.

The positive associations between workload and work-family conflict and burnout suggest that burnout in the educational sector may be related not only to the amount or intensity of work but also to the permeability of the boundaries between occupational and family demands. This is consistent with previous literature showing that sustained overload and interference between work and family roles are common correlates of exhaustion and broader burnout processes among teachers and other helping professionals (37, 38, 48). Substantively, these associations could reflect a process through which demands accumulate across domains and are linked to reduced opportunities for recovery. In the Chilean educational context, where schools have faced post-pandemic pressures, administrative intensification, and growing demands for socioemotional support and coexistence management, this pattern is particularly relevant.

The positive associations of role clarity and social support with educational coexistence are theoretically significant because they suggest that perceptions of coexistence may reflect broader organizational conditions embedded in everyday relationships at school. If educational coexistence is understood as the quality of participation, care, coordination, inclusion, and everyday interaction within the school community, then workers may be more likely to perceive it positively when responsibilities are clear and support is available. In this regard, the findings are consistent with the notion that job resources are related to how workers perceive the social and organizational climate of the school.

Role clarity showed a stronger association with educational coexistence. One possible interpretation is that coexistence initiatives may depend on organizational coordination, explicit responsibilities, and predictable channels of action. When roles are ambiguous, the implementation of coexistence practices may become fragmented, responsibilities may be displaced, and conflict management may depend excessively on improvisation. Conversely, when staff understand what is expected of them and others, they may perceive the institutional environment as more coherent and fairer. This interpretation aligns with the current emphasis in Chilean policy on shifting from reactive and punitive approaches to preventive, formative, and management-oriented ones (30).

Social support was also positively associated with educational coexistence. This result is compatible with the collective nature of schoolwork, where supportive interactions among colleagues and supervisors may coincide with a more favorable working environment, smoother collaboration, and lower interpersonal strain. This pattern is consistent with JD-R formulations in which the presence of resources is linked to broader organizational perceptions and worker well-being (49). However, the coefficient for social support was smaller than that for role clarity.

The negative correlations between demands and resources and between burnout and educational coexistence further suggest that these domains are intertwined. Specifically, workers who reported higher demands tended to report lower perceived resources, a pattern consistent with dynamic approaches to the JD-R model (24). In the present cross-sectional data, these associations may reflect that higher workload and work-family conflict co-occur with less favorable perceptions of social support and role clarity. Likewise, participants reporting higher burnout also tended to report less favorable perceptions of coexistence. Taken together, these correlational patterns are consistent with the view that educational coexistence is embedded in broader psychosocial working conditions rather than functioning as an isolated policy construct.

### 4.1 Practical implications for the Chilean context

The findings may have practical implications for school management and the implementation of educational coexistence policies in Chile. The results are particularly compatible with Chile’s current National Policy on Educational Coexistence, which frames coexistence as a preventive, formative, and organizational matter rather than merely a disciplinary issue. The policy’s emphasis on collective care, inclusion, management, and whole-school coordination is consistent with the observed pattern in which role clarity and social support were associated with more favorable perceptions of coexistence.

On a more specific level, the evidence found in the study suggests the need for differentiated intervention strategies depending on the type of psychosocial factor.

First, to promote role clarity, it is important to properly develop and socialize the organization’s formal structures. Here, it is essential to establish onboarding mechanisms regarding communication channels and authority, clearly defining roles and responsibilities, and establishing effective operational protocols.

Second, it may be beneficial to foster social support, as it is associated not only with individual well-being but also with a stronger sense of community and more favorable organizational perceptions.

Third, given the strength of the associations observed for work-family conflict and workload, efforts to improve work-life balance may not be sufficient on their own. Administrative and structural responses—such as flexible scheduling policies, administrative support, and a review of processes and staffing ratios—could be considered as potentially relevant strategies for addressing workload and work-family strain.

### 4.2 Limitations and future research directions

Despite these results, the study has several limitations. First, the cross-sectional design prevents us from concluding causality or temporal sequence. Although the structural model was specified based on theoretical grounds, alternative directional interpretations are also plausible. Second, all measures were obtained through self-report questionnaires administered in a single survey session, raising the possibility of common method variance and shared response patterns. Third, the educational coexistence instrument was an *ad hoc* measure derived from current Chilean policy. Although preliminary psychometric evidence was favorable, further validation studies are still needed.

Additionally, the non-probability convenience sample restricted to 35 schools in the Atacama region limits the generalizability of the results. Future research should aim to overcome this limitation by extending this line of work to encompass different types of school administration (public, subsidized private, and fee-paying private schools). It has been reported that the institutional context can significantly influence the prevalence of mental health problems and burnout (2, 3, 50). Furthermore, subsequent studies would benefit from stronger evidence on measuring educational coexistence, as well as from longitudinal or multi-informant designs. These designs would allow for a more rigorous and comprehensive examination of the dynamic link between psychosocial working conditions, burnout, and coexistence within the broader Chilean educational system.

## Data Availability

The raw data supporting the conclusions of this article will be made available by the authors, without undue reservation.
